# Synergistic effects of silver nanoparticles in combination with ineffective antibiotics against multidrug resistant *Salmonella typhi*

**DOI:** 10.12669/pjms.40.6.7900

**Published:** 2024-07

**Authors:** Sidra Maqsood, Yasmeen Taj, Luqman Satti, Shaista Bakhat

**Affiliations:** 1 Sidra Maqsood, MBBS, MPhil. Medical Officer Bahawalpur University, Bahawalpur, Pakistan; 2 Yasmeen Taj, MBBS, MPhil, PhD. Professor and Head of Department Pathology, Bahria University Health Sciences, Karachi, Pakistan; 3 Luqman Satti, MBBS, FCPS (Microbiology). Professor PNS Shifa Hospital, Karachi, Pakistan; 4 Shaista Bakhat, MBBS, MPhil. Senior Assistant Professor, Bahria University Health Sciences, Karachi, Pakistan

**Keywords:** Silver-Nanoparticles, *Salmonella*, Multi-drug resistance (MDR), Extensively-drug resistance (XDR)

## Abstract

**Objectives:**

To determine the antimicrobial activity of silver nano-particles(AgNPs) with tetracycline and ampicillin against multi-drug resistance (MDR) and extensively-drug resistance (XDR) *Salmonella typhi*.

**Methods:**

Cross sectional non-probability purposive study was conducted from September, 2021 to May, 2022 at Microbiology department PNS Shifa, Hospital Karachi. Blood cultures of patients suspicious of typhoid fever were collected and incubated in automated Bact/Alert system. Positive cultures were identified on blood and MacConkey and processed by API-10S, confirmed by serotyping (O9 antisera) (SSI Diagnostica’s *Salmonella)*. Antibiotic resistance was done by Kirby-Bauer disk diffusion (Sigma and Rich). MDR and XDR isolates were preserved in Brain Heart Infusion in a volume of 2ml in screw capped bottles at -70°C. Antimicrobial powders (ampicillin and tetracycline (Alfa Aesar) weighed by an electrical weighing balance (OHAUS) to take 1mg of antimicrobial drug. Absorbance spectra of serial concentrations of antibiotics (UV-Vis spectrophotometer (Mole-Qule-) AgNPs (10nm) (nanocomposix) + Antibiotic in (1:1 volume ratio). Conjugation of silver nanoparticles with tetracycline and ampicillin was done by FTIR (thermos scientificThermos ScientificNicolet 50).

**Results:**

Out of 77 isolates, 54 were resistant to ceftriaxone (XDR) and 23 sensitive to ceftriaxone (MDR). All isolates were susceptible to azithromycin and meropenem. Comparison of zone of inhibitions of ampicillin and Amp-AgNPsas and tetracycline with Tet-AgNPs was done. Minimal inhibitory concentration was also done to determine antimicrobial activity.

**Conclusion:**

Significant synergistic inhibitory effects against *Salmonella Typhi* isolates were obtained by combination of tetracycline with silver nano-particles even at low concentration.

## INTRODUCTION

Antimicrobial resistance is a global concern due to the worldwide spread of drug resistant microorganisms also called superbugs. The aggravating factor for the dissemination of resistant microbes is the abuse of antimicrobials substances. WHO established the Global Antimicrobial Resistance and Use Surveillance System (GLASS) in 2015 to fulfill the knowledge gaps and to enforce the work plans at all levels.^1^ World Antimicrobial Awareness Week is also established from 18 to 24 November annually to raise awareness. The slogan used (WAAW) is “Antimicrobials Handle with Care”. The pharmaceutical pipeline for new antimicrobials is presently dry.^2^ Penicillin was the first antibiotic discovered in 1942, the first sign of resistance was noticed in *Staphylococcus* species prompting researchers to discover new antibiotics.^3^ In 1980s, the latest antimicrobial drugs were formulated, after that date no remarkable invention has been reported. The worldwide observations regarding community and nosocomial infections show that we are running out of time. Enteric fever or typhoid fever is a potentially fatal infection caused by *Salmonella enterica* serotype *Typhi*. The word typhoid was derived from ancient Greek word typhos, meaning of this word is ‘cloud of smoke’’ which is chosen to highlight the severity and long lasting neuropsychiatric effects of this disease.

It has been estimated 21 million cases of typhoid fever occur worldwide per year with a mortality rate of 161,000.^4^ various outbreaks of (MDR) strains have been reported for the last two decades with resistance to the first line antibiotics, trimethoprim-sulfamethoxazole, chloramphenicol and ampicillin.^5^ Over the same time period resistance against the fluoroquinolones was observed. Extensively drug resistant (XDR) was documented in Pakistan in the year 2016. XDR strains are only susceptible to azithromycin, tigecycline and carbapenems.^6^

For the last decades nanoparticles have attained a position of utmost importance in the field of medicine and science due to possession of special properties that have vast applications in the biomedical field. Nanoparticles are very small in size ranging from 1nm to 100nm (U.S. Environmental Protection Agency), and cannot be detected by naked eye and ordinary microscopes. They are smaller than the wavelength of visible light (400-700 nm). Silver nanoparticles are vastly employed because of their broad spectrum antimicrobial properties and lower toxicity as compared to other metal nanoparticles.^7^,^8^

Currently silver is utilized to treat wounds, burns, warts in the form of silver nitrate and silver sulfadiazine. The antibacterial mode of action achieved by nanoparticles are that they constantly release silver ions which adhere to the bacterial cell wall and cytoplasmic membrane increasing permeability leading to disruption of the cell.^9^ After penetration they suppress enzyme activity, protein synthesis and generate reactive oxygen species which cause DNA modification and interrupt its replication hence death of the organism.^10^ Bactericidal activity of silver nanoparticles, conjugated with first line antibiotics was determined by using the Kirby Bauer disc diffusion method and minimum inhibitory concentrations were calculated and results were compared with standard drugs.^11^

## METHODS

Blood cultures of patients suspicious of typhoid fever were collected from hospitalized and outpatient at PNS Shifa hospital Karachi from Sep 2021 to May 2022.

In the current study 77, drug-resistant samples were isolated from blood cultures of patients. The sample size was calculated with 16% prevalence, 95% confidence interval and a 5% margin of error (Open epi). Blood culture (Oxoid) (BASINGSTOKE, United Kingdom) specimens were incubated in automated Bact/Alert system (Biomerieux) (Marcy-1’Etoile, France, near Lyon). Positive cultures were identified as on blood and MacConkey (Oxoid) agar further processed by API 10S and serotyping (O9 antisera) (SSI Diagnostica’s *Salmonella*Sero-Quick Group kit). Antibiotic resistance profile was done by Kirby-Bauer disk diffusion method (sigma and rich) according to the CLSI guidelines 2021.

### Ethical Approval

Ethical approval was obtained from Ethical Review Board (ERC) of Bahria University Health Sciences (BUHSCK) reference no 86/2021 on 23^rd^ Dec 2021.

After the identification of MDR and XDRisolates, these were preserved in Brain Heart Infusion (BHI) in a volume of 2ml in screw capped bottles at -70°C temperature. Antimicrobial powders (Ampicillin and tetracycline) (Alfa Aesar 25g packing) were weighed by an electrical weighing balance (OHAUS). Weight of the cuvette was taken and then the antibiotic powder was poured in it and weight of cuvette was minus to take 1mg of antimicrobial drug. Absorbance spectra of serial concentrations of antibiotics using the UV-Vis spectrophotometer (Mole-Qule-On Ingenious) to obtain a range of lamda max for 200-800 nm. First we prepare the Stock solution of antibiotics obtain the lamda max for 200-800 nm range λmax is obtained (357nm). AgNPs (10nm) (nanocomposix) + Antibiotic in (1:1 volume ratio) gently shaken in vortex mixer (VELP) for 30 minutes and kept at room temperature for 24 hours. Quantification of nano-antibiotics was done by centrifugation (HERMLE) of nanoantibiotic solution at 2000 rpm for 30 minutes. Separate the supernatant. Measure the absorbance of supernatant at λmax which we obtained earlier by UV-Vis spectrophotometer. Nanoantibiotic= Total antibiotic - Antibiotic in supernatant.

**Fig.1:**
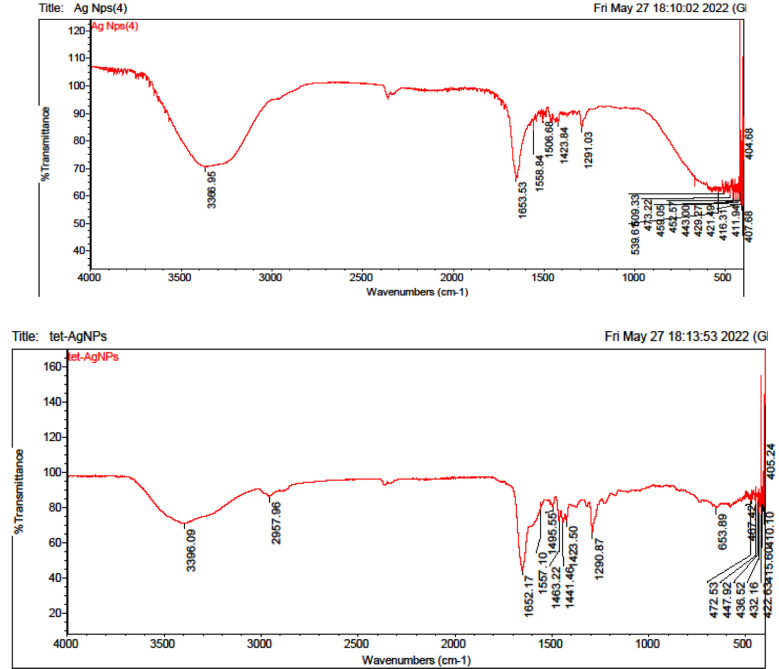
FTIR data for characterization of conjugation of silver nanoparticles with tetracycline.

**Fig.2:**
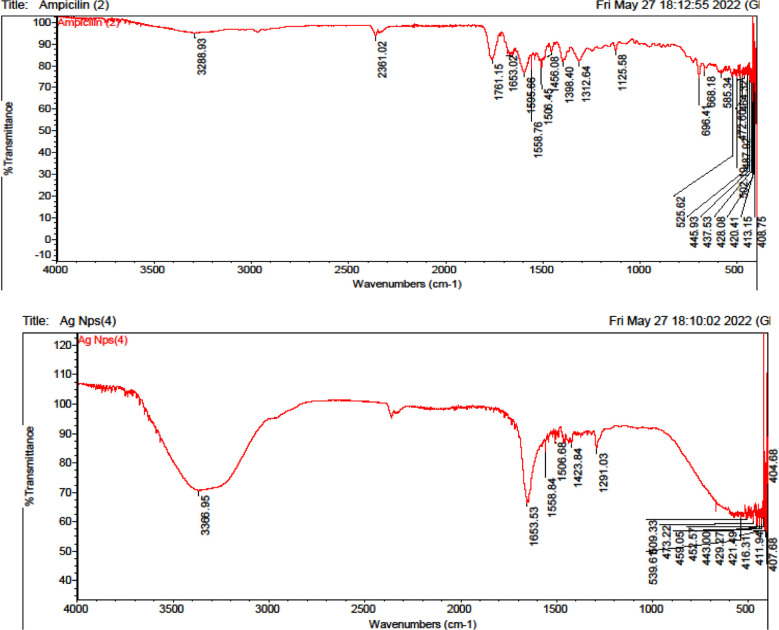
FTIR data for characterization of conjugation of silver nano-particles with ampicillin.

After measuring the value of absorbance of supernatant we measured the concentration of antibiotic at that absorbance by calibration curve. Further characterization of conjugation of silver nanoparticles with tetracycline and ampicillin was done by FTIR (thermoscientificnicolet 50) fourier transform infrared spectrometer with a frequency range of 4000-500 cm-1) NED (University of Engineering and Technology). This spectrometer is based on the principle of fourier transformation of infra-red light after interference. It can perform qualitative and quantitative examination of sample. This technique is highly sensitive and can recognize the functional group from the spectral bands that let us know conjugation has occurred between the nano-material and the bio-molecule that has to be absorbed as per (Fig.1 & Fig.2).

Antimicrobial stock solution of nano-antibiotics was prepared at concentration, ten times higher the concentrations to be tested. Potency = (Assay purity) × (Active fraction)×(1-Water content)Volume (mL) = Actual weight (mg) × Potency (µg/mg) / Desired concentration (µg/ml) Then we made the different dilutions of this stock solution from 2μg/ml to 0.2μg/ml.

Preparation of McFarland solution was done by adding bacterial colonies which were freshly obtained from preserved samples at -70°C by sub-culturing them on a blood agar plate and incubating them at 35 ± 2°C for 24 hours. The McFarland’s standard bacterial suspension of 0.5 turbidity was prepared and it was diluted at 1:20 to yield 5×10^6^CFU/ml. Screening of antimicrobial activity of nano-antibiotics was done by sub-cultured on Mueller-Hinton agar plates.

A small well was formed in the center of plates and 5ug of nanoantibiotics were dropped on the agar plates. Incubate at 37 °C for 24 hours. Zone of inhibition around the nanoantibiotics were measured and compared with the zone of inhibition of antibiotics alone. Minimum inhibitory concentration was done by broth dilution methods. Serial concentrations of nanoantibiotics from 2-0.2µg/ml were pipetted from well 2-11 Wells of column one was taken as growth controls and well no.12 was sterility control. The plate was incubated at 37°C for 24 hours. Results were calculated by visualizing the turbidity in wells of micro titer plate.

### Statistical analysis

Statistical analysis of data was done by using SPSS version 23.0 and MS EXCEL 2010. Results were reported as frequencies for categorical variables i.e. source of specimens. Categorical variables were shown as frequency and percentages. Fischer Exact test was applied to see the significance. P-value ≤0.05 is considered as statistically significant. Graphs were made for different variables.

## RESULTS

Kirby Bauer susceptibility was done with standard strength of antibiotic discs. Out of 77 isolates obtained from blood samples, 54(70.1%) isolates were resistant to ceftriaxone and 23(29.8%) were sensitive to ceftriaxone. All isolates were susceptible to azithromycin and meropenemas shown in Table-I and Table-II.

**Table-I:** Antibiotics Susceptibility of Salmonella Typhito standard antibiotics.

Antibiotics	Frequency Sensitivity	Percentage (%)	Percentage
Ceftriaxone (30µg)	23	29.8	70.1
Azithromycin (30µg)	77	100	0
Meropenem (30µg)	77	100	0
Ampicillin (10µg)	0	0	100
Tetracycline (30µg)	0	0	100
Chloramphenicol (30µg)	0	0	100
Ciprofloxacin (5µg)	0	0	100
Trimethoprim-sulphamethoxazole	0	0	100

**Table-II:** Comparison of zone of inhibitions (in mm) of ampicillin and Amp-AgNPs.

AMP	AMP-AgNPs			Total	P-Value

	2	4	6		
0	1	0	1	2	0.798
2	0	5	2	7	
TOTAL	1	5	3	9	

*Fischer Exact Test was applied to see the significance at P-value ≤0.05.

No growth of test strains was observed after 16-20 hours as mentioned in Table-III. Incubation even at the lowest concentration of 1μg/ml, the dose was decreased ton0.2μg/ml from 2μg/ml i.e. 2, 1.8, 1.6, 1.4, 1.2, 1, 0.8, 0.6, 0.4 and 0.2μg/ml in column 2, 3, 4, 5, 6, 7, 8, 9, 10 and 11 respectively. Results were checked according to (CLSI) guidelines (2021) by the unaided eye (by checking the turbidity). Results were counter checked by sub-culturing inoculum from the lowest concentration wells (well-11) of all rows, on blood agar and incubated at 35 ± 2°C for 24 hours, no growth was observed. The standard value of MICs for tetracycline against this pathogen is 4 μg/ml (according to CLSI 2021). Minimum inhibitory concentration was markedly decreased when tetracycline was conjugated with silver nanoparticles.

**Table-III:** Comparison of zone of inhibitions of tetracycline with Tet-AgNPs.

Tet (30µg)	Tetracycline Ag nanoparticles (5µg)						Total	P-Value

	18	19	20	21	22	23		
0	0	3	14	7	6	0	30	0.006
2	0	1	8	4	4	0	17	
3	0	1	4	4	0	0	9	
4	0	0	5	1	4	2	12	
5	1	0	1	1	0	0	3	
6	0	2	1	1	0	0	4	
7	0	0	0	0	1	0	1	
8	0	0	0	1	0	0	1	
Total	1	7	33	19	15	2	77	

*Fischer Exact Test was applied to see the significance at P-Value ≤0.05.

## DISCUSSION

In Pakistan there has been recent surge in antibiotic resistant cases (MDR) resistant to three antibiotics, ampicillin, trimethoprim-sulfamethoxazole, and chloramphenicol (XDR) resistant to five antibiotics, chloramphenicol, ampicillin, co-trimoxazole, fluoroquinolones, and third-generation cephalosporins).

In this study, we checked the antibacterial activity of antibiotics conjugated with silver nanoparticles against MDR and XDR. It was clearly visible that the combination of tetracycline with AgNPs generated a synergistic inhibitive effect on the growth of *Salmonella typhi*. While the combination of ampicillin and AgNPs did not produce any inhibitory effect on the growth. Antibacterial activity was checked by the agar well diffusion method and minimum inhibitory concentration (MICs) method. The zone of inhibitions by 5µg of tetracycline + AgNPs (Tet-AgNPs) was more than 10mm of the zone of inhibitions when compared to 30µg of tetracycline alone. The MIC value of Tet-AgNPs was 0.2 µg/ml as compared to the standard value of tetracycline i.e. 4 µg/ml. The MIC value was markedly decreased when tetracycline was conjugated with silver nanoparticles. This study showed the highly significant antimicrobial activity of Tet-AgNPs against at the lowest concentration of 0.2µg.Similar to our results Shan MD et al. (2015) demonstrated that the combination of tetracycline with AgNPs generates synergistic antimicrobial effects against *S .typhi murium* while the combination of penicillin and AgNPs do not.^12^ They checked antimicrobial activity by plate counting method at 30minutes and two hours exposure tests. No inhibitive effect was observed with tetracycline alone; however, its combination with AgNPs causes a decrease in bacterial colony forming units. At a concentration of 1.25 µg/ml of tetracycline and they found a decrease is 30% or 30minutes after 30 minutes exposure and 85% after two hours exposure test.

In another study, ampicillin, penicillin, enoxacin, kanamycin, neomycin, and tetracycline were used to explore their synergistic mechanism and showed significant antibacterial effects against MDR *S.typhi* *murium* with all the antibiotics except penicillin and ampicillin. Hussein and Mohammad, (2019)^13^ demonstrated that a combination of tetracycline and biologically synthesized silver nanoparticles had a synergistic inhibitory activity against *S. aureus* and *K. pneumonia* and another study showed antimicrobial activity against MDR *Salmonella* proving that nanoparticles at lower concentration along with cefixime could be a possible alternate to control the MDR pathogens (Kapadia, C et al., 2021).^14^

Although the synergistic effect have been observed before by the combination with tetracycline and other antibiotics against *S. typhi murium* and other bacteria including *E. coli, S.aureus*, *K. pneumonia*, and B. subtilis, according to our knowledge this is the first report on a study with MDR and XDR in Pakistan. No synergistic effect is observed for the β-lactam antibiotic ampicillin when combined with AgNPs. The mechanism of action of polykeptide and β-lactam classes of antibiotics is different in terms of the protein synthesis inhibition. Tetracycline inhibits protein synthesis by binding to the 30S subunit of ribosomes of the bacterium.^15^

Zakir M *et al* identified 46% extensively drug-resistant and 24.5% multi-drug resistant Salmonella typhi.^16^ High fluoroquinolones and cephalosporin resistance was observed in research conducted by Klemm et al^17^ Aside from Pakistan, cases of XDR *S. typhi* have been reported in 23 different countries including Canada, Wong et al.^18^ The multi- country Typhoid Fever Surveillance in Africa Program (TSAP) conducted studies in countries including Burkina Faso, Ethiopia, Ghana, Kenya, Senegal, Madagascar, South Africa, Guinea-Bissau, Tanzania, and Sudan. Both TSAP and the Surveillance of Enteric Fever in Asia Project (SEAP) showed the presence of 52% MDR strains which is figure close to our study.^19^

The phenomena of bacterial genetics exchange of resistance genes among bacterial population has resulted in evolution of multi drug resistant strains. According to the Pakistan National Institute of Health, the city of Karachi witnessed 52 new cases of XDR typhoid fever (Open epi), bringing the total cases reported between January 01, 2017, and Aug 14, 2021, to 15,224. Sensitivity and resistance patterns of our results (Table-I) are in accordance to Fida S et al who reported 71.2% XDR and 21.2% MDR.^20^ Since 2016, a continuous outbreak of XDR typhoid has struck more than 5,000 people in Pakistan, and 11 children in the US who have history of travel to Pakistan. The center of disease control (CDC) listed drug-resistant typhoid fever in the serious threat category. CDC is working with public health partners worldwide, including Pakistani health authorities, to strengthen prevention efforts, including immunization. Globally, around 21 million people are affected, with almost 161,000 deaths reported annually. During 2018-2021, different countries reported cases of typhoid increased from 8,800-1 million till 2021.^21^

This is an in vitro study; further in vivo studies are required to determine the effect of AgNPs conjugated tetracycline for treatment options of multi-drug-resistant and extensively drug-resistant induced typhoid fever.

## CONCLUSION

It is concluded in this study that we can utilize the synergistic therapeutic potentials of nano-particles in particular silver when combined with antibiotics. The synergistic antibacterial effect was observed by combining tetracycline with AgNPs against multi-drug resistant and extensively-drug resistant even at very low concentration.

### Author’s Contribution:

**SM:** Has whole project of MPhil.

**YT:** Conceived, designed, final review and final approval, is responsible for integrity of research.

**LS:** Has facilitated her in bench work and sample collection.

**SB:** Did editing and manuscript writing.
